# Predictors of Clinical Outcome in Primary Progressive and Relapsed Wilms Tumor in a Resource-Limited Setting

**DOI:** 10.7759/cureus.101969

**Published:** 2026-01-21

**Authors:** Muhammad Shahid, Haleema Saeed, Komal Seher, Najma Shaheen, Sajid Ali, Tehreem Zahra

**Affiliations:** 1 Pediatric Oncology, Shaukat Khanum Cancer Hospital and Research Center, Lahore, PAK

**Keywords:** event-free survival, long-term outcome, nephroblastoma, recurrence, wilms tumor

## Abstract

Objective: This study aims to evaluate survival outcomes and identify prognostic factors associated with relapse or treatment-refractory primary progressive Wilms tumor in a resource-limited South Asian setting.

Study design and setting: This is a retrospective cohort study conducted at a single tertiary-care pediatric oncology center in Pakistan.

Materials and methods: Clinical charts of patients aged under 18 years were retrospectively examined and included. Patients who had been diagnosed with relapsed or treatment-refractory primary progressive Wilms tumor during the period between January 2010 and December 2023 were included in the study. The occurrence of disease following full remission after usual therapy was termed relapse, whereas the absence of radiologic or symptomatic response to the first chemotherapy was termed primary progressive disease. The Kaplan-Meier was used to estimate overall survival and event-free survival.

Results: Among 452 children treated for Wilms tumor, 58 (13%) developed relapse or disease progression. Five-year overall survival was 43% (95% confidence interval: 29-57%). Event-free survival (survival without relapse, progression, death, or abandonment) fell to 28% at one year and 20% at two years. At the time of diagnosis, stage 4 disease had a worse survival rate than stage 3 (24% vs 49%).

Conclusions: Presentation at an advanced stage of the disease and the inability to achieve first remission are strong predictors of poor survival.

## Introduction

Wilms tumor is the most prevalent malignant renal neoplasm in the pediatric population [1]. Multimodal management (surgery, risk-adapted chemotherapy, and well-planned radiotherapy) has shown excellent results in high-income populations, achieving event-free survival rates near 90% [2]. In contrast, in low- and middle-income countries (LMICs), the rate of relapse is between 30% and 40% targeted by multimodal treatments, which explains the markedly lower outcomes of this malignant disease [3].

Various elements lower the survival rates in LMICs, such as late presentation with metastatic disease, discontinuation of treatment, and a lack of a supportive-care infrastructure [4]. As a result, treating relapsed disease in resource-limited settings is especially challenging and is quite often linked to poor outcomes [5]. The post-relapse survival depends on a number of variables that interact, including the stage of the disease at earlier diagnosis, the nature of the underlying tumor histopathology, the nature of the first-line chemotherapy, the anatomical location of the recurrence, the amount of disease burden at the time of relapse, and the disease-free interval before recurrence [6].

There is a lack of evidence detailing the management approaches and outcomes of relapsed and treatment-refractory progressive Wilms tumor in LMICs, especially in Pakistan. A single-center study from Pakistan reported an event-free survival rate of 63%, with relapse in 15% of cases. Furthermore, a systematic review and meta-analysis demonstrated that treatment-related mortality among children with cancer was significantly higher in LMICs at 14.2% (95% CI 9.65-18.73), compared with 4.47% (95% CI 3.42-5.53) in upper-middle-income countries (p < 0.0001) [7].

Contemporary salvage strategies increasingly rely on risk-stratified approaches, whereby patients with higher-risk disease receive more intensive treatment regimens. Identifying and addressing predictors of outcome in children with primary progressive or relapsed Wilms tumor is therefore essential to improving survival [8,9]. In this context, the present study aims to outline the clinical features and determine factors associated with treatment outcomes among patients with relapsed or treatment-refractory primary progressive Wilms tumor treated at our institution [10].

In resource-limited settings, outcomes following relapse or primary progressive Wilms tumor remain poorly characterized, and data on failure patterns and treatment-related mortality are scarce. The primary objective of this study was to evaluate survival outcomes, including overall survival and event-free survival, among children with relapsed or treatment-refractory primary progressive Wilms tumor treated at a tertiary pediatric oncology center in Pakistan. Secondary objectives were to identify clinical and treatment-related factors associated with adverse outcomes, describe patterns and timing of treatment failure, and quantify the impact of treatment-related mortality. By clearly defining these outcomes, this study aims to provide context-specific evidence to inform risk stratification, supportive care strategies, and future salvage treatment approaches in similar resource-limited settings.

## Materials and methods

This study was conducted as a retrospective, cohort-based observational study. It was hospital-based and carried out at a single tertiary-level pediatric oncology facility, the Department of Pediatric Oncology at Shaukat Khanum Memorial Cancer Hospital and Research Centre, Lahore, Pakistan. The study period spanned from January 2010 through December 2023.

A formal sample size calculation was performed to confirm that the available cohort was sufficient to detect meaningful differences in survival outcomes; however, all patients meeting eligibility criteria during the study period were ultimately included. The calculation was based on a previously reported relapse rate of approximately 15% for Wilms tumor, as documented in a comprehensive review [11]. Using a 95% confidence level and a margin of error of 5%, the single population proportion formula $N = \left[\frac{Z_{\alpha/2} + Z_{\beta}}{C(r)}\right]^2 + 3$ was applied, where Z = 1.96, p = 0.15, and d = 0.05, yielding an initial sample size estimate of 196. Given the finite and known population of children treated for Wilms tumor at the center during the study period (N = 452), a finite population correction was applied using the formula $n_f = \frac{n}{1 + \frac{n - 1}{N}}$, resulting in an adjusted sample size of 58 patients. All eligible cases were included consecutively to achieve this target.

The definitions of relapse and primary progressive disease were defined. For our study, relapse was defined as the reappearance of Wilms tumor after the patient had achieved complete remission following completion of initial therapy, confirmed by clinical, radiological, or histopathological evidence. On the other hand, primary progressive disease was defined as persistent or worsening disease during first-line treatment, with failure to achieve an initial remission, as evidenced by clinical deterioration or radiological progression despite standard therapy.

The non-probability consecutive sampling was used. The study sample included children under 18 years of age with the diagnosis of Wilms tumor with radiological or histopathological signs of relapse or primary progressive disease, and the treatment was conducted within the study institution. Patients were excluded if medical records were incomplete or unavailable, if treatment was terminated during primary treatment, or if follow-up was lost before relapse or progression could be established.

Data collection started after receiving approval of the Institutional Review Board of Shaukat Khanum Memorial Cancer Hospital and Research Centre (approval number: EX-50-03-20-02-A1, dated 26 July 2024). The information was retrieved retrospectively from the hospital's electronic medical record system. Diagnosis of Wilms tumor was done by the use of histopathology and imaging studies in line with International Society of Pediatric Oncology (SIOP) diagnostic criteria.

The records were created using an extraction proforma. Sociodemographic variables involved age at diagnosis and sex, whereas the clinical variables comprised tumor laterality, histological subtype, tumor stage at diagnosis, first-line treatment, response to first-line treatment, time to relapse, recurrence location, type of salvage therapy used, second remission, survival status, and cause of death. Clinical documentation and death summaries were used to support the assessment of survival outcomes and causes of death.

Possible confounding variables, including disease stage, histology, and treatment relationship, were considered in stratified analyses. Selection bias was reduced since all eligible patients were enrolled during the specified period, and information bias was minimized by comparing data between various record sources. Informed consent was obtained during the treatment process, and the selected procedures ensured the confidentiality and anonymity of the research. This was done in accordance with the principles of the Declaration of Helsinki. SIOP institutional standards and guidelines were used to determine treatment procedures, such as chemotherapy regimens and surgical procedures.

First-line chemotherapy regimens were administered according to contemporaneous SIOP-based institutional protocols in use during the study period, typically incorporating vincristine, actinomycin D, and doxorubicin for intermediate- and high-risk disease, with dosing adjusted for body surface area and organ function. Salvage chemotherapy regimens were selected based on prior treatment exposure, disease extent at relapse or progression, treatment tolerance, and clinician judgment. Commonly used regimens included carboplatin and etoposide alternating with cyclophosphamide and doxorubicin, as well as ifosfamide, carboplatin, and etoposide (ICE) or cyclophosphamide-based combinations. Exact dosing schedules varied over time and were individualized, reflecting real-world practice rather than a single standardized protocol.

SPSS Statistics version 26 (IBM Corp. Released 2019. IBM SPSS Statistics for Windows, Version 26.0. Armonk, NY: IBM Corp.) was used to analyze the data. Age and time to relapse were continuous variables, and normality was assessed using the Shapiro-Wilk test. Means and standard deviations were used to summarize normally distributed data, and interquartile ranges and medians were used to define non-normally distributed variables. Frequencies and percentages of categorical variables were reported, including sex, disease stage, histological subtype, relapse pattern, and survival status.

Categorical variables were evaluated by means of the chi-square test. For continuous variables, the independent t-test was used for normal distributions, and the Mann-Whitney U test was used for non-normal distributions. Kaplan-Meier was used to estimate overall and event-free survival, and the comparison of survival curves was performed using the log-rank test. Overall survival was defined as the time from relapse or progression to the end of life; event-free survival was defined as the time from relapse or progression to another relapse, disease progression, or death. Treatment abandonment was defined as failure to attend scheduled follow-up visits for more than four weeks during curative therapy.

Confounding influences were further addressed through subgroup and stratified analyses. Bias was minimized through consistent operational definitions, standardized data extraction procedures, and comprehensive inclusion of eligible cases. Statistical significance was determined using a p-value threshold of less than 0.05.

Data quality was ensured through standardized data extraction using a predefined pro forma and cross-verification of key variables across electronic medical records, imaging reports, and pathology documentation. Missing data were minimal and handled using complete-case analysis; no data imputation was performed. Patients with substantially incomplete records were excluded at the eligibility stage.

## Results

During the study period, 452 children were treated for Wilms tumor. Fifty-eight patients (13%) developed relapse (24 of 58) or treatment-refractory progressive disease (34 of 58) and were included in the analysis. The median age at diagnosis was 3.0 years (range: 0.7-10.0 years). Most children presented with advanced disease, as only 16 of 58 (27.6%) had stage I or II disease at diagnosis. Forty patients (69%) received treatment with curative intent after relapse, while 18 patients (31%) received palliative care due to advanced disease or poor treatment tolerance.

Favorable histology was seen in 50 patients (86%), and unfavorable histology in eight patients (14%). No significant difference in survival was observed between histologic groups (p > 0.05). All patients had undergone nephrectomy during initial treatment. Anthracyclines were used in 38 patients (66%), and radiotherapy in 25 patients (43%). Baseline characteristics were similar between patients with primary progressive disease and those with relapse.

Relapse or primary progression occurred early in most patients. The mean time to treatment failure was 4.2 months, and 91% of events occurred within the first year. Primary progressive disease was observed in 34 patients (59%), while 24 patients (41%) relapsed after achieving remission. Metastatic relapse was most common and occurred in 29 patients (50%). Mixed local and metastatic disease was seen in 18 patients (31%), while isolated local relapse occurred in 11 patients (19%).

Among patients treated with curative intent, salvage chemotherapy regimens varied. Seventeen patients (43%) received carboplatin and etoposide alternating with cyclophosphamide and doxorubicin. Twenty-one patients (52%) received ICE or CYCE regimens. Two patients (5%) received other regimens. Eighteen patients (31%) received palliative chemotherapy or supportive care only.

The event-free survival was always lower than overall survival, with 28% and 20% survival at one year and two years, respectively, indicating that there were early adverse events, especially disease progression, death, and discontinuation of treatment.

The leading cause of mortality was disease progression, as 32 of 58 (55%) of all deaths were caused by it; next came infectious complications, primarily sepsis in 21 of 58 (36%), and cardiac toxicity in five of 58 (9%). It is quite noteworthy that approximately half of the fatalities were a result of causes other than direct tumor progression.

Disease stage had a statistically significant impact on survival at the initial stage (p = 0.028). Stage I-II, III, and IV-V patients had 60%, 49%, and 24% survival rates at 5 years, respectively. Radiotherapy given at the first treatment was associated with lower survival, but this was not statistically significant (p = 0.053). The influence of stage at relapse also emerged as a factor affecting survival, though the differences were not significant. Phenotypes of relapse showed that local-only events were associated with the most favorable outcomes, whereas combined local and metastatic events were associated with the worst survival.

Time to treatment failure was a significant factor (p = 0.041). Patients who had primary progressive disease had worse survival rates in comparison to those who had relapse after remission. Previous anthracycline use did not affect post-relapse survival (p = 0.834).

Table 1 presents the demographic profile and baseline clinical characteristics of children diagnosed with relapsed or refractory Wilms tumor, including age, sex, disease stage at initial diagnosis, histological subtype, and details of first-line treatment.

**Table 1:** Demographic profile and baseline clinical characteristics of patients with relapsed or primary progressive Wilms tumor (n = 58)

Characteristic	n (%)
Total patients with relapse or progression	58 (13%)
Age at initial diagnosis, median (range), years	3.0 (0.7-10.0)
Sex	
Male	35 (60%)
Female	23 (40%)
Initial disease stage	
Stage I	6 (10%)
Stage II	10 (17%)
Stage III	23 (39%)
Stage IV	14 (24%)
Stage V (bilateral)	5 (9%)
Histology at presentation	
Favorable	50 (86%)
Unfavorable	8 (14%)

Table 2 describes the treatment exposure and relapse-related characteristics, including time to relapse, type of relapse, pattern of disease recurrence, and stage at relapse.

**Table 2:** Initial treatment exposure and relapse characteristics SD: standard deviation

Variable	n (%)
Initial treatment received	
Anthracycline-based chemotherapy	38 (66%)
Radiation therapy	25 (43%)
Nephrectomy	58 (100%)
Relapse characteristics	
Time to relapse, mean ± SD (months)	4.2 ± 7.61
Relapse within 12 months	53 (91%)
Primary progressive disease	34 (59%)
True relapse after remission	24 (41%)
Stage at relapse	
Localized	11 (19%)
Metastatic	29 (50%)
Combined local and metastatic	18 (31%)

Table 3 presents patient outcomes at the last follow-up, including survival status, treatment intent at relapse, treatment abandonment, and causes of death.

**Table 3:** Treatment intent at relapse and outcomes at last follow-up

Outcome	n (%)
Treatment intent at relapse	
Curative	40 (69%)
Palliative only	18 (31%)
Status at last follow-up	
Alive without disease	10 (17%)
Alive with disease (palliative care)	9 (16%)
Deceased	31 (53%)
Treatment abandonment	8 (14%)

Table 4 summarizes the univariate analysis examining variables associated with overall survival, including disease stage at initial presentation, exposure to radiotherapy, stage at relapse, timing of treatment failure, and prior use of anthracycline-based chemotherapy.

**Table 4:** Univariate analysis of factors associated with overall survival CI: confidence interval

Variable	n (%)	5-year overall survival (%)	95% CI	p-value
Initial disease stage				0.028
Stage I-II	16 (27.6%)	60	38-82	
Stage III	23 (39.7%)	49	27-71	
Stage IV-V	19 (32.8%)	24	6-42	
Prior radiation therapy				0.053
No	33 (56.9%)	72	56-88	
Yes	25 (43.1%)	47	27-67	
Stage at relapse				0.304
Localized	11 (19.0%)	70	43-97	
Metastatic	29 (50.0%)	50	31-69	
Combined	17 (29.3%)	39	16-62	
Timing of treatment failure				0.041
True relapse	24 (41.4%)	62	42-82	
Primary progression	34 (58.6%)	51	34-68	
Prior anthracycline exposure				0.834
No	20 (34.5%)	58	36-80	
Yes	38 (65.5%)	56	40-72	

**Figure 1:**
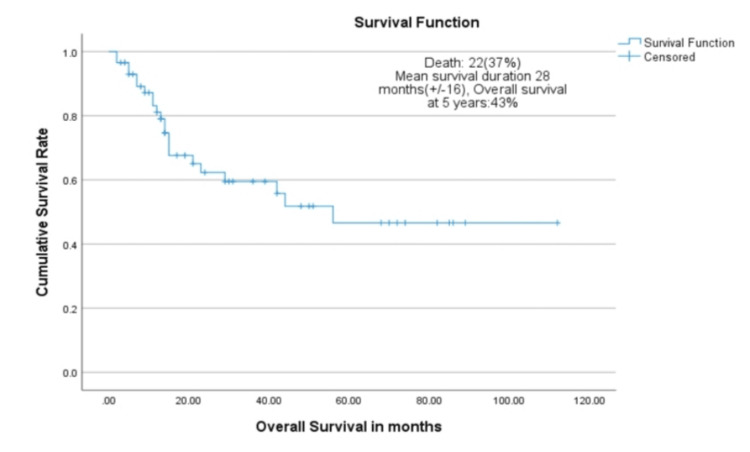
Kaplan-Meier curve demonstrating overall survival for 58 patients with relapsed or primary progressive Wilms tumor

Figure 1 shows a Kaplan-Meier curve demonstrating overall survival for 58 patients with relapsed or primary progressive Wilms tumor. The curve shows five-year overall survival of 43% (95% CI: 29-57%) with a mean survival duration of 52.3 months (95% CI: 38.8-65.7 months).

**Figure 2:**
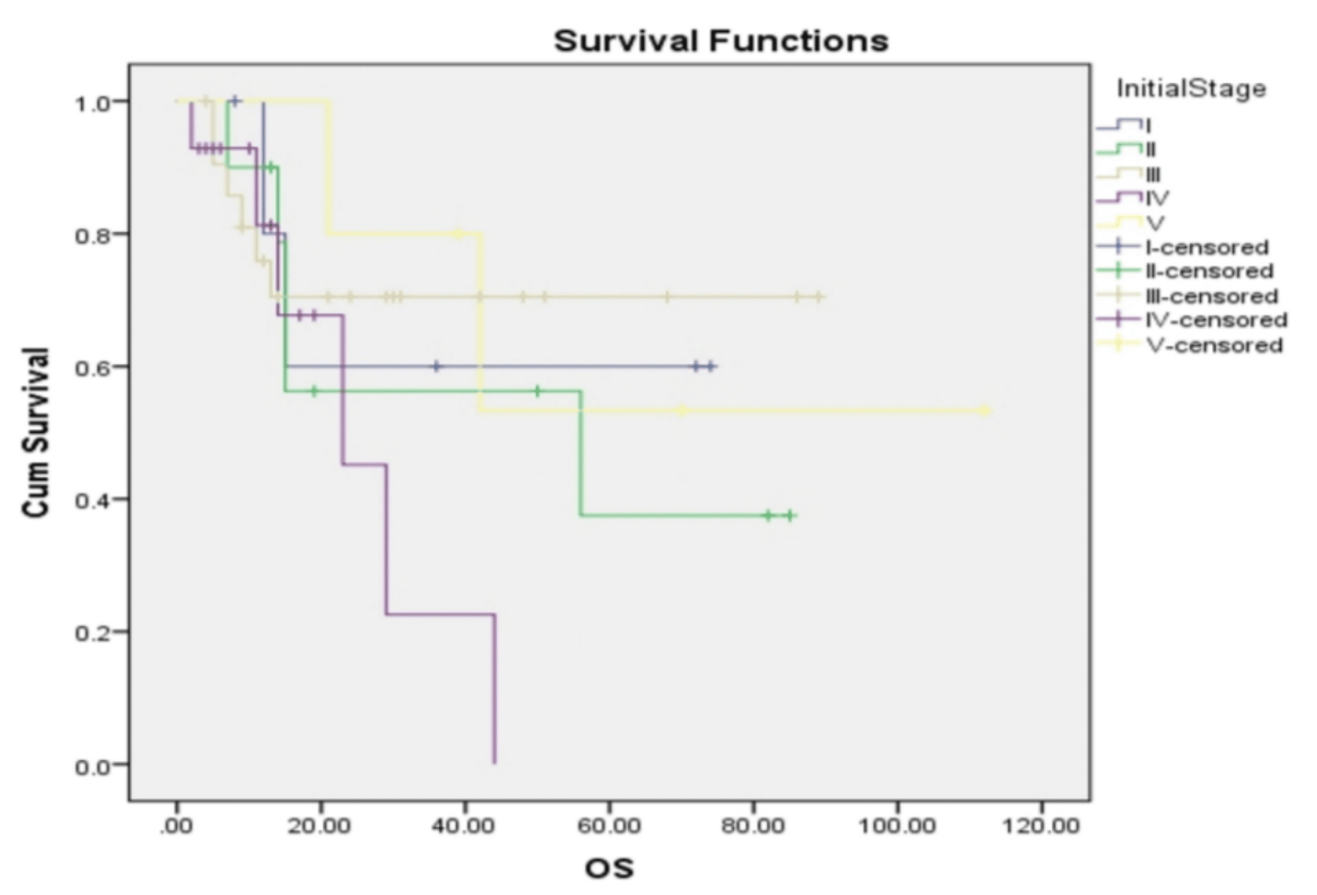
Kaplan-Meier curves stratified by initial disease stage at diagnosis OS: overall survival

Figure 2 shows Kaplan-Meier curves stratified by initial disease stage at diagnosis. Patients with stage IV-V disease demonstrated significantly worse outcomes (five-year overall survival 24%, 95% CI: 6-42%) compared to stage I-II (60%, 95% CI: 38-82%) and stage III (49%, 95% CI: 27-71%), p = 0.028 by log-rank test.

**Figure 3:**
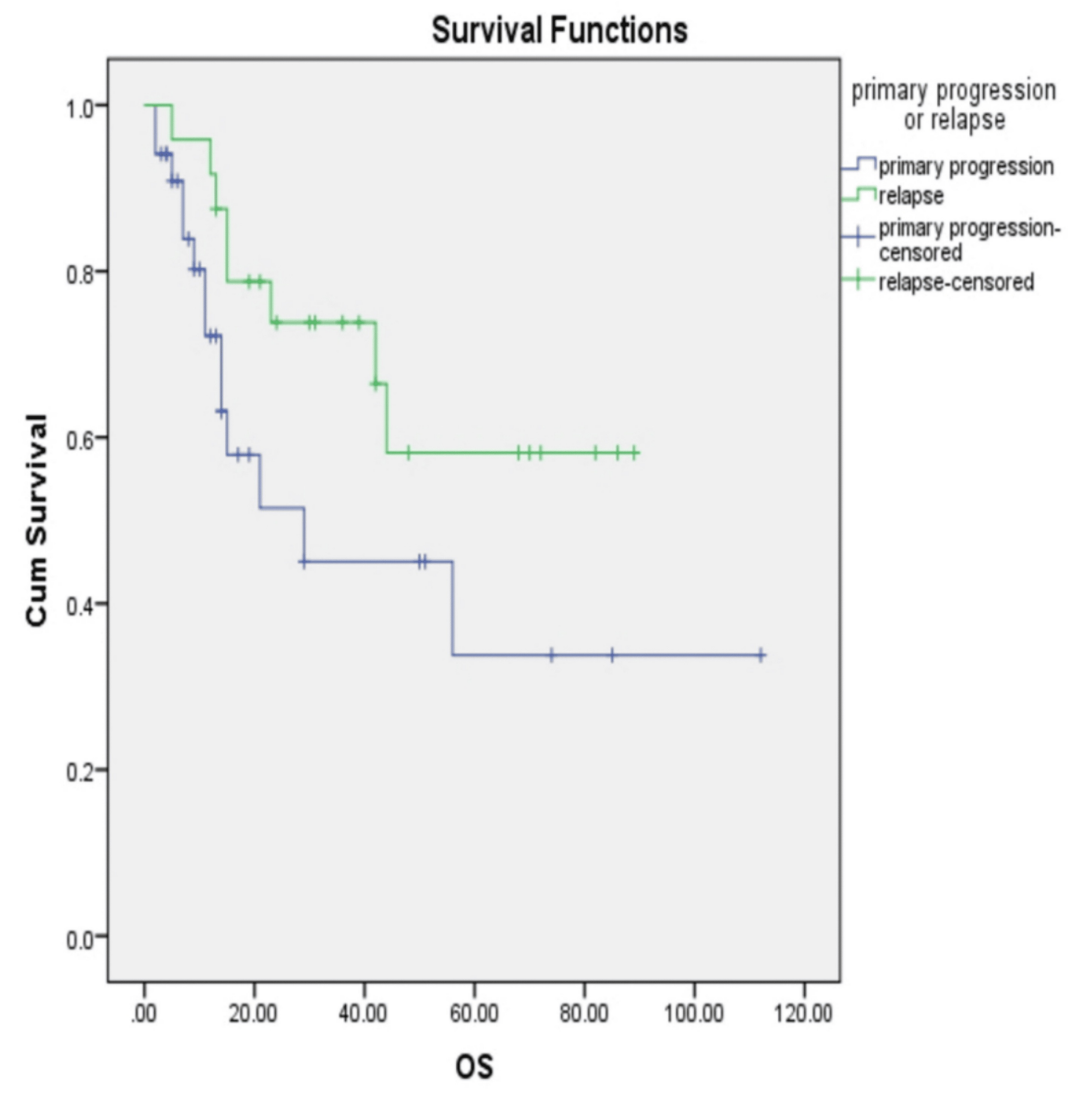
Kaplan-Meier curves comparing primary progressive disease (51% overall survival) versus true relapse after remission OS: overall survival

Figure 3 shows Kaplan-Meier curves comparing primary progressive disease (51% overall survival) versus true relapse after remission (62% overall survival), p = 0.041. Primary progression, defined as failure to achieve initial remission, is associated with significantly worse outcomes.

**Figure 4:**
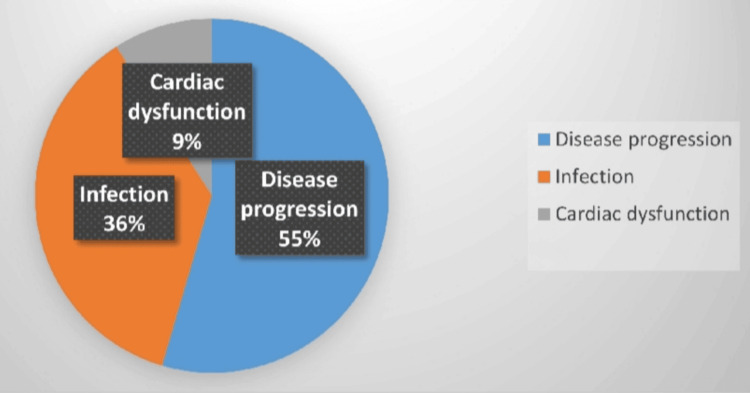
Distribution of causes of death among 31 deceased patients

Figure 4 shows the distribution of causes of death among 31 deceased patients. Disease progression accounted for 17 deaths (55%), sepsis/infection-related complications for 11 deaths (36%), and cardiac dysfunction for three deaths (9%). Non-disease-related deaths comprised 45% of total mortality, highlighting the substantial treatment-related mortality burden in this resource-limited setting.

## Discussion

The findings of this study demonstrate a five-year overall survival rate of 43%, with event-free survival remaining notably low at both the one- and two-year time points. These outcomes are substantially inferior to those documented in high-income countries [12]. Nevertheless, they are broadly consistent with reports originating from other LMICs, where similar challenges in treatment delivery and supportive care persist. In contrast, survival rates of 70% to 80% have been reported in selected cohorts of patients with relapsed Wilms tumor treated in well-resourced settings [13]. Such favorable outcomes are typically observed among patients who experience localized relapse, have a prolonged disease-free interval, and have been exposed to a limited-intensity prior therapy. These factors collectively contribute to improved responsiveness to salvage treatment [14].

Most children in this cohort presented with advanced disease at initial diagnosis. This significantly reduced the chance of a cure after relapse. Late presentation, limited referral systems, and financial constraints are common in low-resource settings. These factors often lead to delayed treatment and increase the risk of treatment abandonment [15].

The initial disease stage was the strongest predictor of outcome [16]. Patients with stage IV disease had the poorest survival [17]. This finding is consistent with published data showing that the advanced stage is linked to aggressive tumor behavior and poor response to therapy. Such tumors are more likely to relapse early and respond poorly to salvage treatment [18].

Prior exposure to radiotherapy was associated with a trend toward worse survival [19]. This likely reflects the use of radiation in patients with more advanced or high-risk disease. Radiation-related toxicity may also limit the ability to deliver intensive salvage chemotherapy. Larger studies are needed to clarify this association.

In patients with a localized relapse, survival was better than in patients with a metastatic relapse or combined relapse [20]. Even though this difference did not reach statistical significance, the trend remained apparent. In cases of localized relapse, surgical resection and combination therapy may be used, and in metastatic relapse, the disease burden is higher, and response to therapy is low.

Individuals who had a relapse following attainment of remission exhibited better outcomes as compared to those who had primary progressive disease [21]. Loss of the ability to attain an initial remission is probably an indicator of aggressive tumor biology or a weak response to first-line therapy. Such a subgroup is thus a significantly high-risk population.

One of the salient observations arising from the current analysis relates to the significant morbidity in terms of treatment-related deaths. About 50% of total deaths were independent of tumor growth. Out of those causes that arose not as a result of disease, sepsis became the most significant cause of death, followed by anthracycline-induced cardiac dysfunction. These preventable deaths can be reduced significantly by improving infection-prevention measures, prompt intervention and control of sepsis, and strict cardiac monitoring during and after treatment [22].

Abandonment of treatment remained a significant setback to the overall results. Although the abandonment rate in this cohort was lower than that reported in some other LMIC studies, it still had a substantial impact on survival gains. This problem has several underlying factors that are interrelated, such as distance to treatment centers, cultural and social beliefs, poverty, and weak psychosocial support systems. All of these burdens are aggravated in the situation of relapsed disease, where treatment regimens are stricter, treatment duration is prolonged, and uncertainty of prognosis can also demotivate continued participation in care. This study represents the largest single-center report of relapsed Wilms tumor from Pakistan. The long follow-up period strengthens the survival estimates. Detailed documentation of treatment-related deaths adds important information that is often underreported in LMIC studies.

Limitations of the study

The retrospective design is a limitation. Selection and survival biases may be present. The single-center nature limits generalizability. A small sample size prevented multivariable analysis and led to wide confidence intervals. Molecular risk factors could not be assessed, as such testing was not routinely available.

## Conclusions

Relapsed and primary progressive Wilms tumors showed poor outcomes in this setting. Advanced stage at diagnosis and failure to achieve first remission were strong predictors of poor survival. Treatment-related mortality was high and remains a major concern. Improved supportive care, better infection control, and careful cardiac monitoring are essential. Multicenter studies are needed to strengthen evidence and to develop risk-adapted salvage strategies. Early integration of palliative care should be considered for patients with multiple poor prognostic factors.
